# Comparison of DNA extraction kits for PCR-DGGE analysis of human intestinal microbial communities from fecal specimens

**DOI:** 10.1186/1475-2891-9-23

**Published:** 2010-05-22

**Authors:** Merlin W Ariefdjohan, Dennis A Savaiano, Cindy H Nakatsu

**Affiliations:** 1 Department of Foods and Nutrition, Purdue University, 700 West State St., West Lafayette, IN 47907-2059, USA; 2 Department of Agronomy, Purdue University, 915 West State St., West Lafayette, IN 47907-2054, USA

## Abstract

**Background:**

The influence of diet on intestinal microflora has been investigated mainly using conventional microbiological approaches. Although these studies have advanced knowledge on human intestinal microflora, it is imperative that new methods are applied to facilitate scientific progress. Culture-independent molecular fingerprinting method of Polymerase Chain Reaction and Denaturing Gradient Gel Electrophoresis (PCR-DGGE) has been used to study microbial communities in a variety of environmental samples. However, these protocols must be optimized prior to their application in order to enhance the quality and accuracy of downstream analyses. In this study, the relative efficacy of four commercial DNA extraction kits (Mobio Ultra Clean^® ^Fecal DNA Isolation Kit, M; QIAamp^® ^DNA Stool Mini Kit, Q; FastDNA^® ^SPIN Kit, FSp; FastDNA^® ^SPIN Kit for Soil, FSo) were evaluated. Further, PCR-DGGE technique was also assessed for its feasibility in detecting differences in human intestinal bacterial fingerprint profiles.

**Method:**

Total DNA was extracted from varying weights of human fecal specimens using four different kits, followed by PCR amplification of bacterial 16S rRNA genes, and DGGE separation of the amplicons.

**Results:**

Regardless of kit, maximum DNA yield was obtained using 10 to 50 mg (wet wt) of fecal specimens and similar DGGE profiles were obtained. However, kits FSp and FSo extracted significantly larger amounts of DNA per g dry fecal specimens and produced more bands on their DGGE profiles than kits M and Q due to their use of bead-containing lysing matrix and vigorous shaking step. DGGE of 16S rRNA gene PCR products was suitable for capturing the profiles of human intestinal microbial community and enabled rapid comparative assessment of inter- and intra-subject differences.

**Conclusion:**

We conclude that extraction kits that incorporated bead-containing lysing matrix and vigorous shaking produced high quality DNA from human fecal specimens (10 to 50 mg, wet wt) that can be resolved as bacterial community fingerprints using PCR-DGGE technique. Subsequently, PCR-DGGE technique can be applied for studying variations in human intestinal microbial communities.

## Background

The microbial community colonizing the human gastrointestinal (GI) tract is diverse [1] and plays an important role in digestion, production of essential vitamins, as well as protecting the GI tract from pathogen colonization [2,3]. Dietary approaches such as the ingestion of non-digestible oligosaccharides (prebiotics) and fermented food products containing live culture (probiotics) have been speculated to confer health benefits by enhancing the growth of beneficial intestinal bacteria [4]. The influence of diet on intestinal microflora has been largely studied using conventional microbiological techniques. Many limitations are associated with these techniques, but a significant drawback comes from their reliance on the identification of appropriate growth nutrients and conditions. Estimates indicate that only 20 - 40% [5] of the total intestinal microflora can be cultured using standard laboratory protocols. This factor is further complicated by the need to ensure viability of the intestinal bacteria in the samples, many of which are anaerobic [6]. Thus, new analytical tools that can be applied in clinical studies are needed to overcome these limitations.

In the past two decades, molecular techniques based on 16S rRNA gene and other genetic markers have been developed to analyze bacterial communities in environmental samples (e.g., lakes, soil) [7,8]. These methods have an advantage over conventional microbiological techniques because the presence of viable bacteria are not required [9]. Further, the use of genetic materials allows detection of species that cannot be cultured using standard laboratory protocols. Thus, data derived from these molecular techniques provide a more complete analysis of the bacterial communities. A molecular fingerprinting technique that combines PCR-amplification of 16S rRNA gene and separation of amplicons using Denaturing Gradient Gel Electrophoresis (PCR-DGGE) has produced successful results in monitoring variations in microbial community in various environmental samples [7,8]. However, its application in clinical studies has been limited [10-12].

The analytical success of molecular techniques, including PCR-DGGE, is greatly affected by its reliance on cell lysis efficiency and the quality of DNA recovered from the environmental samples. DNA isolation methods that contribute to insufficient cell lysis or shearing of DNA may cause bias in PCR amplification [13,14]. Inhibitors in fecal specimens, such as bile salts and complex polysaccharides, can create similar problems [13,15]. In addition, the amount of fecal specimen used in the extraction process affects extraction efficacy [14]. Hence, it is important that upstream protocols (e.g., DNA extraction) are optimized in order to obtain accurate results. Various commercial DNA extraction kits have been developed to simplify and speed up the extraction process. However, the relative efficacy of these kits and the optimum range of sample weight for extraction need further evaluation.

The goal of this study was to compare the relative efficacy of four commercial DNA extraction kits (Mobio Ultra Clean^® ^Fecal DNA Isolation Kit; QIAamp^® ^DNA Stool Mini Kit; FastDNA^® ^SPIN Kit; FastDNA^® ^SPIN Kit for Soil) in extracting bacterial genomic DNA from human fecal specimens. These kits were selected due to their availability, cost, ease of use, popularity, and differences in cell lysis methods. Although these kits have been tested separately by different researchers on various biological samples [14,16-18], our study further extends the knowledge by direct comparison and application to PCR-DGGE. Specifically, this study evaluates the influence of cell lysis techniques, fecal specimen weight used in extraction, and fecal dry matter content on DNA yield. Ultimately, we aim to demonstrate that optimized conditions for extraction maximizes DNA yield obtained from fecal specimens. Subsequently, the DNA can be used for PCR-DGGE to evaluate variations in human intestinal microbial communities in clinical studies.

## Methods

### Subjects

Healthy volunteers aged 20 - 30 yr (n = 4) were recruited from a college community. Volunteers were non-smokers, did not have any food allergies, had not used antibiotics for the past 6 months, and did not have any history of GI diseases (stomach ulcers, colon cancer, recent bouts of diarrhea, acid reflux disease, heartburn). Female participants were not pregnant or lactating at the time of study. Protocols were approved by the Committee on the Use of Human Research Subjects at Purdue University, West Lafayette, IN.

### Fecal Collection and Fecal Dry Weight Determination

Fecal specimens were collected from each volunteer once a month for a total of 4 specimens per volunteer, which were then stored at -20°C prior to being analyzed. To determine fecal moisture content, frozen specimens were thawed at 4°C. Then, approximately 0.5 g (wet wt) of each fecal specimen was placed in a vacuum dryer for 3 d and re-weighed. Percent fecal dry weight was calculated using the following formula:

To ensure sample homogeneity, remaining fecal specimens were diluted with sterile water (1:2 wt/vol) and then kneaded in separate sterile plastic bags using a stomacher at high speed for approximately 5 min. A sub-sample was aliquoted for DNA extraction and the remainder stored at -20°C.

### DNA Extraction and Quantification

The following four commercial DNA extraction kits were evaluated:

**M **- Mobio Ultra Clean^® ^Fecal DNA Isolation Kit (MO BIO Laboratories, Inc., Carlsbad, CA)

**Q **- QIAamp^® ^DNA Stool Mini Kit (Qiagen Inc., Valencia, CA)

**FSp **- FastDNA^® ^SPIN Kit (MP Biomedicals, Irvine, CA)

**FSo **- FastDNA^® ^SPIN Kit for Soil (MP Biomedicals, Irvine, CA)

Three variables were tested for kit extraction efficacy: i) ratio of water to dry matter content of fecal specimens, ii) wet fecal specimen weight used for extraction, and iii) cell lysis method. Fecal specimens submitted by the subjects varied in their percent dry matter content, which may be correlated with the microbial concentration and subsequently the quantity of DNA extracted from the fecal specimens. This would make it difficult to use a standardized method in clinical studies. To investigate this, three fecal specimens with differing dry matter content (26%, 35%, and 41%) from different individuals were selected for extraction.

Protocols supplied with the kits recommended different amounts of starting materials for extraction (Table 1). Preliminary experiments showed that extracting from fecal specimens above 200 mg (i.e., 300 mg and 500 mg) were not feasible. This amount overloaded the purification matrix and caused the filter to rupture. Thus, five specimen weights (10, 25, 50, 100, and 200 mg (wet wt)) were selected to evaluate the efficacy of the DNA extraction kits.

**Table 1:** Comparison of recommended DNA extraction protocols based on technical booklets included with respective extraction kits.

Extraction kit/Steps	M^1^	Q^1^	FSp^1^	FSo^1^
**Fecal wt (mg)**	250 - 1000	180 - 220	200	500

**Beads**	Unknown beads	None	Ceramic + garnet	Ceramic + silica

**Cell lysis and homogenization**	Flat bed vortexer (10 min)	Centrifuge (14,000 rpm, 1 min)	Fast Prep^® ^Instrument (speed 6.0, 40 s)	Fast Prep^® ^Instrument (speed 5.5; 30 s)

**Adsorption of inhibitors**	IRS^**2 **^solution	InhibitEx tablet	None listed	None listed

**Approximate time to completion^3^**	45 to 60 min	60 to 80 min	45 to 60 min	60 to 80 min

**Average cost of kit^4^**	$176.00	$181.00	$344.20	$240.45

^**1**^M, Mobio Ultra Clean^® ^Fecal DNA Isolation Kit; Q, QIAamp^® ^DNA Stool Mini Kit; FSp, FastDNA^® ^SPIN Kit; FSo, FastDNA^® ^SPIN Kit for Soil

^**2**^Abbreviations are not defined by manufacturers due to being proprietary in nature

^**3**^Approximate time to complete DNA extraction for 12 samples using kits M, FSp, and FSo, but only 6 to 8 samples for kit Q (due to using table-top vortexer for shaking as listed in the protocol)

^**4**^Prices as of May 2010 (based on 50 preparations for kits M, Q, and FSo; 100 preparations for kit FSp)

Additional experiments with modifications to the standard protocol were conducted to determine whether the use of vigorous shaking, specifically using the FastPrep^® ^Instrument, was the key determinant in influencing DNA yield. The two kits, M and Q, that did not use a FastPrep^® ^Instrument, were tested by homogenizing 25 mg of a fecal specimen (26.3% dry matter) in their respective lysing matrices using the FastPrep^® ^Instrument for 30 seconds at a speed setting of 5.5. The lysis solution Q did not contain any beads and none were added. Supernatant from each mixture was then processed using subsequent steps in the respective protocols of kits M and Q.

DNA yield was quantified by fluorometric analysis (Picofluor, Turner BioSystems, Sunnyvale, CA) using calf thymus DNA as a standard. Values for DNA yield were normalized based on the dry weight of the respective fecal specimen. DNA quality was evaluated using gel electrophoresis on 0.8% agarose gels stained with ethidium bromide, visualized on a UV transilluminator and photographed (UVP BioImaging system, UVP LLC, Upland, CA).

### PCR-DGGE Analysis

PCR-DGGE technique was used to evaluate the microbial community profiles from the respective fecal specimens. Bacterial 16S rRNA gene V3 region was amplified by PCR using primers PRBA338F (5' **CGC CCG CGC GCG GCG GGC GGG GCG GGG GCA CGG GGG **GAC TCC TAC GGG AGG CAG CAG 3'; GC-clamp is in boldface) [19] and PRUN518R (5' ATTA CCG CGG CTG CTGG 3') [20]. The GC-clamp, which is a sequence that is rich in guanine and cytosine, is added to the 5' end of the forward or reverse primer in order to prevent DNA from being completely denatured into single strands. Subsequently, this improves band resolution in denaturing gels. The final PCR reaction mixture (50 μl total volume) contained 5 μl of 10× PCR Buffer, 4 μl of 25 mM MgCl_2_, 0.4 μl of 100 mM deoxynucleotide triphosphate mixture, 2.5 μl of 20 mg/ml bovine serum albumin, 0.75 μl of each primer (at 25 μM), 1 μl of 5 U/μl Taq polymerase, and 1 μl DNA template (approximately 10 ng/μl). The amplification condition was 94°C for 5 min (initial denaturation), followed by 30 cycles of denaturation at 92°C for 30 sec, annealing at 55°C for 30 sec, and extension at 72°C for 30 sec. A final extension step was carried out at 72°C for 15 min. Presence of PCR products were confirmed by electrophoresis on 1.5% agarose gels stained with ethidium bromide in 1× TAE buffer using Lambda DNA-Hind III Digest (New England BioLabs, Inc., Ipswich, MA) as a molecular weight standard. Gels were visualized on a UV transilluminator and photographed (UVP BioImaging system, UVP LLC, Upland, CA).

PCR amplicons were separated using DGGE, which was conducted using the DCode™ Universal Mutation Detection System (Bio-Rad Laboratories, Hercules, CA), with slight modifications to the method previously described by Muyzer et al. [21]. Equal masses of PCR products were separated on 8% (wt/vol) polyacrylamide gels (40% acrylamide/bis solution, 37.5:1; Bio-Rad Laboratories, Hercules, CA) in 1× TAE (40 mM Tris, 20 mM Acetate, 1.0 mM Na_2_-EDTA) using denaturing gradient ranges of 35 to 50%, 45 to 60%, and 35 to 60%, where a 100% denaturant contained 7 M urea and 40% (vol/vol) deionized formamide. Electrophoresis was performed at 50 V for 10 min, then at 200 V for 5.5 hr. Electrophoresis buffer (1× TAE) was maintained throughout at 60°C. Gels were then stained using SYBR Green I nucleic acid stain (Cambrex Bio Science, Rockland, ME), visualized on a UV transilluminator, and photographed (UVP BioImaging system, UVP LLC, Upland, CA).

### Analysis of Bacterial DGGE Banding Profiles and Sequencing

Similarities between banding patterns in the DGGE profile were calculated based on the presence and absence of bands and expressed as a similarity coefficient. In this study, the Dice similarity coefficient was used to calculate pairwise comparisons of the DGGE fingerprint profiles obtained from the four DNA extraction kits. This similarity coefficient is calculated based on the following formula: D_sc _= [2*j*/(*a*+*b*)], where *a *= number of DGGE bands in lane 1, *b *= number of DGGE bands in lane 2, and *j *= number of common DGGE bands in lane 1 and lane 2, and D_sc _= 1 indicates identical profiles [22]. Dendrograms showing clustering according to the similarity of banding patterns between samples were constructed by the unweighted pair group method of arithmetic averages (UPGMA) [23] using BioNumerics software (BioNumerics, Applied Maths, Inc., Austin, TX).

### Statistical Analysis

All extractions were performed in triplicate to account for analytical variability. Means of DNA yield were analyzed using SAS (version 9.1; SAS Institute, Cary, NC) by one-way and two-way ANOVA. Differences between treatments were grouped by Tukey test. Data were expressed as means ± SE. Differences were considered as significant when P was < 0.05.

## Results

### Relative Efficacy of DNA Extraction Methods

**Figure 1:**
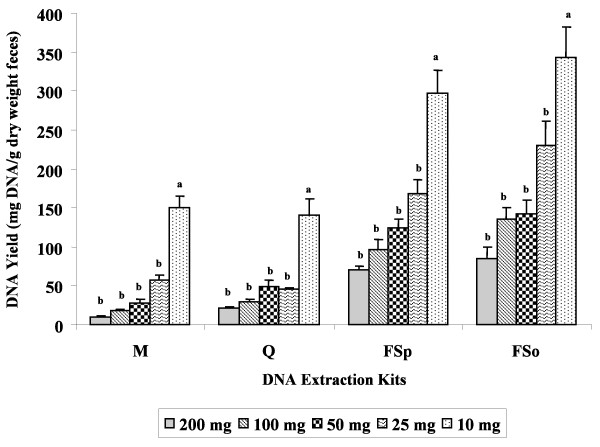
**Average DNA yield obtained using the four commercial kits as influenced by fecal specimen weights**. DNA was extracted from 200, 100, 50, 25, and 10 mg of human fecal specimens (n = 45/kit), using Mobio Ultra Clean^® ^Fecal DNA Isolation Kit (M), QIAamp^® ^DNA Stool Mini Kit (Q), FastDNA^® ^SPIN Kit (FSp), and FastDNA^® ^SPIN Kit for Soil (FSo). Values for DNA yield were normalized based on the dry weight of the respective fecal specimen. Means with different letter designation are significantly different (comparisons within each extraction kit; P < 0.05).

**Figure 2:**
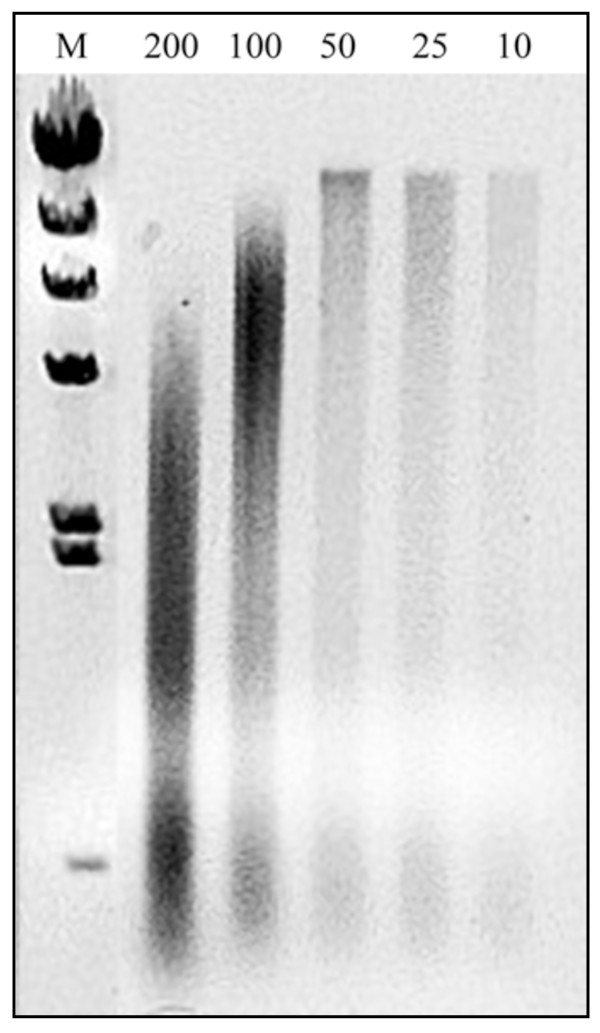
**Quality of DNA extracted from varying weights of fecal specimens using kit FSo**. DNA was extracted from 200, 100, 50, 25, and 10 mg of human fecal specimens using FastDNA^® ^SPIN Kit for Soil (FSo). Note shearing of DNA in extractions using higher fecal weights.

Despite differences in extraction variables (weight of fecal specimens used in the extraction and fecal dry matter contents) and protocols (Table 1), the four commercial kits evaluated were found to be successful in extracting DNA from human fecal specimens (Table 2). However, kits FSo and FSp produced approximately three times more DNA than kits M and Q when quantities were normalized by fecal dry weight. DNA yield from kits Q and M did not significantly differ from each other. A negative correlation was observed between the amount of fecal weight used for extraction and DNA yield (Figure 1). Fecal sample weights of 100 and 200 mg, but not 10, 25 and 50 mg, produced large quantities of sheared DNA (Figure 2). Hence, the optimum DNA yield (i.e., in terms of DNA quantity and quality) was obtained by extracting 10 to 50 mg of fecal specimen (wet wt).

**Table 2:** Average DNA yield obtained using the four commercial DNA extraction kits^1^.

DNA extraction kit^1^	DNA yield (mg DNA/g dry wt feces)^3^
M	52.4 ± 14.5^**b**^

Q	57.0 ± 22.6^**b**^

FSp	151.3 ± 47.1^**a**^

FSo	187.2 ± 69.4^**a**^

The following extractions accounted for various fecal specimen weights (10, 25, 50, 100, and 200 mg) and DNA yield was normalized by percent fecal dry matter (26%, 35%, and 41%)^2^.

^**1**^M, Mobio Ultra Clean^® ^Fecal DNA Isolation Kit; Q, QIAamp^® ^DNA Stool Mini Kit; FSp, FastDNA^® ^SPIN Kit; FSo, FastDNA^® ^SPIN Kit for Soil

^**2**^Values of DNA yield were based on n = 45/DNA extraction method and were normalized based on the dry weight of the respective fecal sample

^**3**^Treatment groups with different letters indicate significant differences between groups (P < 0.05). Values are means ± SE.

**Figure 3:**
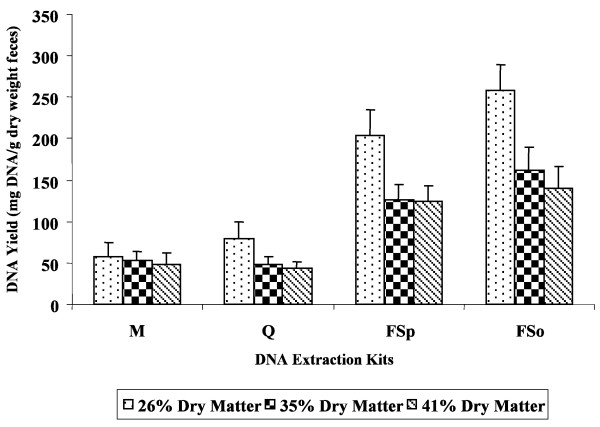
**Average DNA yield obtained using the four commercial kits as influenced by fecal dry matter**. The percent dry matter in the human fecal specimens were 26%, 35%, and 41% (n = 45/kit). Values for DNA yield were normalized based on the dry weight of the respective fecal sample. DNA from the fecal specimens were extracted using Mobio Ultra Clean^® ^Fecal DNA Isolation Kit (M), QIAamp^® ^DNA Stool Mini Kit (Q), FastDNA^® ^SPIN Kit (FSp), and FastDNA^® ^SPIN Kit for Soil (FSo).

The percentage of dry matter in the fecal specimen also influenced DNA yield (Figure 3). Kits Q, FSp, and FSo produced significantly higher amount of DNA from fecal specimens containing the lowest percent of dry matter (26%), followed by 35% and 41%. In contrast, DNA yield obtained from kit M did not significantly differ regardless of the percent dry matter in the fecal specimens.

**Figure 4:**
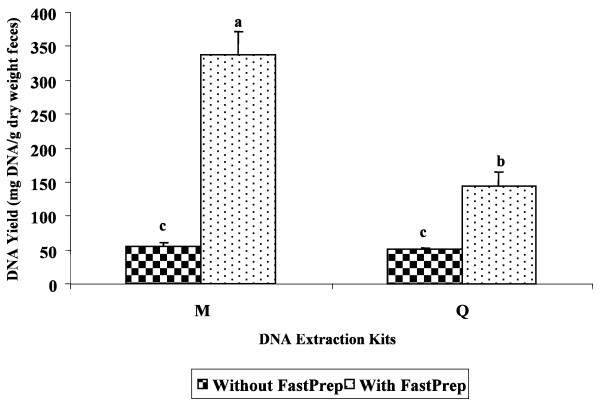
**Average DNA yield obtained using kits M and Q**. Comparison was made on the average DNA yield of these kits with and without the addition of vigorous mixing using the FastPrep^® ^Instrument (n = 3/kit; M, Mobio Ultra Clean^® ^Fecal DNA Isolation Kit; Q, QIAamp^® ^DNA Stool Mini Kit). Values for DNA yield were normalized based on the dry weight of the respective fecal sample. Means with different letter designation are significantly different (P < 0.05).

DNA yield from kit M was significantly improved by incorporating a vigorous shaking step using the FastPrep^® ^Instrument instead of vortexing (as suggested by the manufacturer's protocol) (Figure 4). Although kit Q did not contain beads, which may have contributed to its significant lower DNA yield than kit M, the additional step of vigorous shaking using the FastPrep^® ^Instrument contributed to a higher DNA yield in kit Q than extraction performed without this step (Figure 4).

### Comparative Analysis of DGGE Fingerprint Profiles

**Figure 5:**
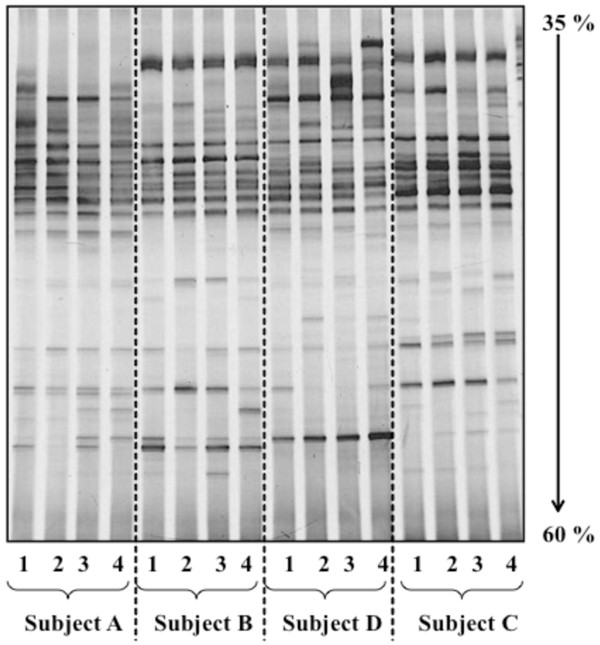
**A comparison of DGGE profiles of PCR amplified bacterial 16S rRNA gene**. DNA was extracted using FastDNA^® ^SPIN Kit for Soil (FSo) using 25 mg of fecal specimens collected from four human subjects (Subject A, B, C, and D; n = 4 for each subject). Bacterial fingerprint profile is based on 35 to 60% DGGE gel gradient. Lane 1 to 4 show bacterial fingerprint profile of consecutive fecal samples collected from each subject.

**Figure 6:**
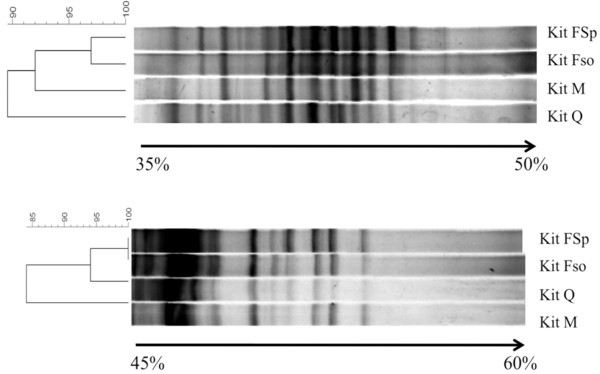
**Dendrograms generated from PCR-DGGE profiles obtained from DNA extracted using the four commercial kits**. The dendrograms were based on Unweighted Pair Group Method with Arithmetic Averages (UPGMA). Dendrogram A is based on 35 to 50% DGGE gradient gel and dendrogram B is based on 45 to 60% DGGE gradient gel.

The broad range of bands comprising community profiles were visible across a 35 to 60% gradient, and intra- and inter-subject variations were readily observed (Figure 5). However, there were many bands in the upper part of the DGGE gel that were not sufficiently resolved to describe differences. As such, it is imperative that PCR amplicons from the same set of samples be separated using DGGE gels of multiple gradients in order to better distinguish common and less common bands. Consequently, this practice will increase scoring accuracy and may also facilitate the detection of a broader profile of bacterial communities. The differences in band resolution were best illustrated in comparisons of profiles of the same sample using different DNA extraction kits. Theoretically, all profiles should have been identical since DNA was extracted from the same homogenized source. Using a DGGE gel gradient of 35 to 50%, the Dice similarity coefficient of bacterial community profiles from DNA extracted using the four kits ranged from 0.88 to 0.97. There was little difference in profiles generated from DNA extracted using kits FSo and FSp (similarity coefficient of 0.97). On the other hand, the profile from kit Q was the least similar to the others, whereby its Dice similarity coefficient was 0.88 when compared to kit FSo (Figure 6; Profile A). Using a 45 to 60% gradient gel, similarity coefficients ranged from 0.82 to 1.0. Profiles from kits FSo and FSp were identical (similarity coefficient value of 1). However, using this gradient, the profile from kit M was the least similar to the others with coefficient value of 0.82 when compared kits FSo and FSp (Figure 6; Profile B). In addition to the differences observed when different denaturing gradients were used, these results suggest that kits Q and M were not able to extract DNA from all the bacteria in the fecal specimen. This is likely due to less efficient cell lysis by kits Q and M.

## Discussion

Our data indicate that optimum DNA yield can be obtained from human fecal specimens using commercial extraction kits that specifically incorporate a lysing matrix containing beads and an instrument that produces a vigorous shaking motion (bead-beating system). DNA extraction kits Fso and FSp that we evaluated produced a higher quantity of DNA than kits M and Q that did not incorporate bead beating. Our findings using kits FSp and M were consistent with those of Scupham et al. [17], whereby kit FSp produced a tenfold higher amounts of DNA than kit M. Further, quantity and quality of DNA extract was influenced by the amount of fecal specimen used. Data from this study indicate that 10 to 50 mg of fecal specimen (wet wt) was optimal for maximum DNA extraction. This produced high quality DNA that can be amplified using PCR and separated by DGGE for comparisons of microbial fingerprint profiles. Collectively, these observations demonstrate that optimal DNA yield from human fecal specimens is achieved by thorough cell lysis as facilitated by bead-beating. This observation is also consistently seen in other samples, such as soil [24,25]. Higher extraction efficiency allows for better recovery of DNA from an environmental sample resulting in a more comprehensive and complete profile of the bacterial community within the sample [26-29]. On the other hand, poor DNA extraction may lead to DGGE fingerprint profiles that are not representative of the bacterial community. Higher DNA yield also increases recovery of DNA from the bacterial community member in a sample and thus, increasing chances of detecting rare species [17].

The quantity of DNA extracted from fecal specimens is primarily influenced by mechanical cell lysis. However, the motion of a table-top vortex, even when fitted with a specially designed vibrating tray (as the one used in kit M), was not sufficiently vigorous for extracting optimum DNA yield, unlike the FastPrep^® ^Instrument. Kit Q, which did not include a bead-containing lysing matrix and relied solely on chemical lysis, produced lower DNA yield than kit M even after being shaken vigorously by the FastPrep^® ^Instrument. Li et al. [18] showed that kit Q produced higher quantities of DNA than kit FSo. But modifications in the extraction protocol may have contributed to their results. Specifically, fecal specimens were pre-incubated at 70°C and 95°C, zirconia/silica beads were added into the lysing matrix of kit Q, and then the mixture was processed using a FastPrep^® ^Instrument. Conversely, all DNA extractions performed in our study closely followed the protocols supplied in the respective kits. Our data suggest that kit FSo can be used to obtain optimum DNA yield from human fecal specimens, such that modifications to its protocol are not necessary.

The amount of fecal material used for extraction and its moisture content were also observed to influence the final DNA yield. Smaller amounts of specimen used in extraction resulted in higher DNA yield relative to sample mass, which could be attributed to better contact between beads, lysing buffer, and fecal specimen. We also observed that purification filters ruptured due to larger masses of fecal specimens used in the extraction. Consequently, this would lower DNA purity and potentially inhibit downstream analyses.

A negative correlation was observed between percent fecal dry matter and DNA yield. The majority of fecal matter dry weight has been found to be of bacterial origin and its contribution remains relatively constant [30,31]. However, a substantial increase in consumption of certain dietary fibers has been found to reduce the bacterial fraction of fecal dry matter [30] and could account for the lower DNA yield. More information of subject diet is needed to further explore this option. It is also possible that fecal specimens with higher percentage of dry matter may have more fibrous materials that interfered with cell lysis. To overcome this problem some have suggested that DNA yield can be increased by prolonging bead-beating time [27]. However, such treatment may shear DNA creating bias during PCR amplification and subsequently producing inaccurate fingerprint profiles [32]. Further studies are needed to draw conclusions about fecal moisture content and final DNA yield.

Purity of extracted DNA is of importance for downstream molecular work and researchers often prefer to use kits that ensure removal of inhibitors. Kits M and Q, but not kits FSp and FSo, list the addition of specific chemicals to adsorb PCR inhibitors. However, the bacterial 16S rRNA gene region was successfully amplified using DNA extracted by the four kits evaluated and similar DGGE banding profiles were produced. Thus, either PCR inhibitors did not seem to limit the quality of DNA extracted using kits FSp and FSo, or a proprietary reagent contained in these kits was equally efficient in removing inhibitors. The quality of Taq polymerase, the addition of bovine serum albumin in PCR, and thermal cycler setting have also been shown to improve amplification of PCR products from DNA extracted from clinical samples [33,34]. A combination of these factors may have contributed to our success in getting high quality PCR products that produce satisfactory DGGE fingerprinting profiles.

## Conclusions

Our data indicate that optimum DNA yield from human fecal specimens can only be obtained using extraction systems that incorporate bead-containing lysing matrix and an instrument that affords a vigorous shaking motion (bead-beating system). DNA quantity was also significantly improved when 10 to 50 mg of fecal specimens (wet wt) were used in the extraction procedure since these amounts did not overload the extraction matrices. We also provided evidence that PCR-DGGE could be performed with the DNA and the resulting bacterial fingerprint profiles showed within and between subject differences in intestinal microbial communities. This illustrates how PCR-DGGE could be an important tool for clinical studies, such as those evaluating changes in intestinal microbial community in response to dietary treatments.

## List of Abbreviations

ANOVA: Analysis of Variance; DGGE: Denaturing Gradient Gel Electrophoresis; DNA: Deoxyribonucleic Acid; FSo: FastDNA^® ^SPIN Kit for Soil; FSp: FastDNA^® ^SPIN Kit; M: Mobio Ultra Clean^® ^Fecal DNA Isolation Kit; PCR: Polymerase Chain Reaction; Q: QIAamp^® ^DNA Stool Mini Kit; SE: Standard Error; UPGMA: Unweighted Pair Group Method of Arithmetic Averages

## Competing interests

The authors declare that they have no competing interests.

## Authors' contributions

- MWA was involved experimental design, recruited subjects, collected specimens, performed experiments including data analyses, and wrote the first draft of the manuscript.

- DAS was involved in experimental design, data analyses and interpretation, and edited drafts of the manuscript.

- CHN provided input into experimental design, data analyses and interpretation, other important technical support associated with the experiments, as well as editing drafts of the manuscript.

- All authors have read and approved the final manuscript (version revised and submitted by 20 May 2010)
